# Prenatal stress, hormones, and fetal brain development: gender differences

**DOI:** 10.1007/s42000-025-00696-6

**Published:** 2025-07-25

**Authors:** Vasiliki Georgousopoulou, Aspasia Serdari, Georgios Manomenidis, Catherine Dacou-Voutetakis

**Affiliations:** 1 Department of Nursing, Democritus University of Thrace, Alexandroupolis, Greece; 2 Department of Child and Adolescent Psychiatry, Democritus University of Thrace, Alexandroupolis, Greece; 3 Division of Endocrinology, Metabolism and Diabetes, 1 st Department of Pediatrics, Medical School, National and Kapodistrian University of Athens, Athens, Greece

**Keywords:** Prenatal stress, Fetal brain development, Hypothalamic–pituitary–adrenal axis, Neurodevelopment, Sex differences, Epigenetics, Maternal health, Early intervention

## Abstract

Prenatal stress is increasingly recognized as a critical factor influencing fetal brain development, with potential long-term risks for neurodevelopmental and psychiatric disorders. This review explores the effects of prenatal stress on the fetal brain, with particular emphasis on sex differences and the interplay between genetic, epigenetic, and environmental factors. The activation of the hypothalamic–pituitary–adrenal (HPA) axis in response to maternal stress leads to increased levels of glucocorticoids, which can cross the placenta and alter fetal brain structure and function. Key regions affected include the prefrontal cortex, hippocampus, and amygdala, which are crucial for emotional regulation, memory, and cognitive function. Sex differences play a significant role in fetal stress responses, with males exhibiting greater vulnerability to adverse prenatal conditions. Studies suggest that male fetuses exposed to high maternal cortisol levels exhibit alterations in brain connectivity, increased amygdala volume, and heightened stress reactivity, whereas female fetuses may show adaptive mechanisms that confer resilience. Additionally, maternal nutritional status influences fetal neurodevelopment, with both undernutrition and obesity contributing to structural and functional changes in the fetal brain. Advances in neuroimaging techniques, including high-resolution magnetic resonance imaging (MRI) and placental diffusion imaging have provided valuable insights into how prenatal stress affects fetal brain connectivity. Epigenetic modifications, such as DNA methylation and histone modification, are implicated in the long-term programming of stress responses. Furthermore, early-life interventions, including maternal social support and mindfulness-based therapies, may mitigate the adverse effects of prenatal stress on offspring neurodevelopment. Given the profound impact of prenatal stress on fetal brain development, early identification of at-risk populations and the implementation of targeted interventions are crucial. Future research should integrate findings from neuroscience, genetics, and endocrinology to develop preventive strategies that improve neurodevelopmental outcomes and reduce the burden of stress-related disorders in later life.

## Introduction

Stress is defined as a state of mental tension and worry, triggered by real or perceived adverse circumstances, which helps to maintain homeostasis. This response is primarily mediated by activation of the hypothalamic–pituitary–adrenal (HPA) axis and the subsequent release of key hormones including corticotropin-releasing hormone, adrenocorticotropic hormone, antidiuretic hormone, catecholamines, and anti-inflammatory peptides [1]. The extent and type of stress response as well as the subsequent effect depend on multiple factors, the target organ and timing of stress exposure being playing a critical role [2]. Due to its delicate and prolonged developmental trajectory, the brain is particularly vulnerable to prenatal stress [3]. Numerous studies suggest that maternal stress during pregnancy may alter fetal brain development, primarily affecting diencephalic structures involved in mood regulation, stress metabolism, and vascular control [4, 5]. Within this context, advances in neuroimaging techniques, including high-resolution magnetic resonance imaging (MRI), proton magnetic resonance spectroscopy, and placental diffusion imaging, have significantly enhanced our ability to assess fetal brain alterations associated with intrauterine stress exposure [4, 5].

Although several recent reviews have addressed the effects of prenatal stress on neurodevelopment, the present article offers a unique contribution by emphasizing the sex-specific impact of stress. It integrates findings across diverse disciplines including endocrinology, neuroscience, epigenetics, and developmental psychology providing a comprehensive and multidimensional synthesis of current evidence.

The present review aims to synthesize recent findings on fetal brain development, differentiating typical and atypical trajectories and identifying potential preventive or early therapeutic interventions.

## Methods

To ensure a comprehensive and balanced overview of the literature on prenatal stress and fetal brain development with an emphasis on sex differences we conducted a structured narrative review of peer-reviewed publications. The literature search was performed between January and March 2024 using the following electronic databases: PubMed, Scopus, and Web of Science. The following keywords and Boolean operators were used in various combinations: *“prenatal stress,” “maternal stress,” “fetal brain development,” “sex differences,” “HPA axis,” “epigenetics,” “telomeres,” “glucocorticoids,” “fetal programming,” “mini-puberty,”* and *“neurodevelopment.”* The inclusion criteria for article selection were as follows: original research studies (human and relevant animal models), systematic reviews, meta-analyses, expert reviews, studies published in English between 2000 and 2024, articles focusing on the neurodevelopmental outcomes of prenatal stress exposure, studies examining sex-dependent effects, epigenetic or hormonal mechanisms, neuroimaging correlates, and articles addressing protective or resilience factors such as maternal support, postnatal environment, or interventions. Exclusion criteria included the following: Publications not available in full text, articles focusing solely on postnatal stress or unrelated psychiatric outcomes, and case reports or opinion pieces lacking empirical data. In total, 30 articles were selected and analyzed in depth based on relevance and scientific rigor. Representative studies included large-scale cohort analyses and neuroimaging and mechanistic investigations into mini-puberty.

## Prenatal stress and brain development

The intrauterine period is a critical window for brain development. Emerging evidence underscores the fact that modifiable factors, such as maternal environment and nutrition, can influence neurodevelopmental outcomes. Specifically, epidemiological and experimental studies demonstrate that early-life exposure to adverse environments is associated with long-term neurodevelopmental and psychiatric risks [6, 7]. The study of Bleker et al. suggests that prenatal undernutrition is associated with decreased physical function in later life in men but not in women. Furthermore, the data of the latter work provide evidence for the hypothesis that prenatal undernutrition may lead to an accelerated aging process in humans. Moreover, exposure to raised concentrations of cortisol early in gestation was associated with a slower rate of development over the first year and lower mental development scores at 12 months. The data suggest that maternal cortisol and anxiety have programming influences on the developing fetus [6, 7]. Below, key factors influencing prenatal brain development are discussed.

## Neurohormonal regulation of brain development

Sex hormones play a pivotal role in fetal brain organization with androgens and estrogens influencing brain structure through genomic and non-genomic pathways, modulating neuroprotection and behavioral outcomes. Additionally, testosterone exposure during critical developmental periods has been linked to structural differences in the amygdala and prefrontal cortex, potentially influencing emotional regulation and cognitive function [8, 9].

## Nutritional influences on fetal brain development

Bleker et al. provided compelling evidence that fetal exposure to famine during the first trimester of pregnancy, specifically, during the Dutch Hunger Winter of World War II, was associated with reduced brain volume in adulthood [6]. The effects were sex-specific, with males exhibiting greater susceptibility, likely due to genetic and epigenetic influences. Additional studies confirm that maternal undernutrition affects fetal brain connectivity and increases the risk of neurodevelopmental disorders. The prenatal period is a time particularly vulnerable to environmental effects, however, with distinct gender differences [10]. Important environmental risk factors are maternal mild pre-eclampsia, asthma, overweight or obesity status, and gestational diabetes. For example, high maternal body mass index (BMI) during pregnancy is associated with increased risk for a large number of somatic disorders as well as cognitive and mental health problems in the offspring across the lifespan and possibly during the prenatal period. Salzwedel et al. have shown that maternal adiposity influences neonatal brain functional connectivity (FC). The FC differences can be found as early as during the neonatal period, strongly suggesting early-life programming of the neonatal brain in response to maternal obesity [11, 12]. While severe maternal undernutrition is uncommon today, even mild fetal exposure to unbalanced maternal diets can have lasting effects on stress responses and cognitive function in adulthood [13].

Conversely, maternal obesity is associated with increased risks of impaired neurodevelopment, executive dysfunction, and neuropsychiatric disorders. Follow-up of pregnant women has demonstrated that higher maternal BMI is linked to altered fetal brain FC, particularly in circuits governing cognitive control and emotional regulation [11, 14]. Studies employing fetal MRI revealed that fetuses of obese mothers had significantly larger cortical plates and whole cerebellum volumes compared to those of mothers with BMI (18.5–24.9 kg/m^2^) [15]. Additionally, maternal metabolic conditions, such as gestational diabetes and hyperglycemia, have been linked to increased inflammation and oxidative stress in the fetal brain, potentially leading to neurocognitive impairments. Sex differences in the prevalence, age of onset, and etiology of many psychiatric disorders are well documented. On average, males exhibit larger total brain volume than females. Moreover, regional differences in brain structure—particularly in the amygdala, hippocampus, and insula—have been associated with the sex-biased manifestation of neuropsychiatric conditions. Regional sex differences in volume and tissue density include the amygdale, hippocampus, and insula, areas known to be implicated in sex-biased neuropsychiatric disorders [16, 17].

## Epigenetic mechanisms and prenatal stress

Epigenetic modifications play a crucial role in fetal programming. DNA methylation, histone modifications, and non-coding RNA expression patterns have been linked to neurodevelopmental plasticity. Recent research suggests that prenatal stress may induce stable epigenetic alterations, affecting stress regulation and cognitive outcomes. Additionally, early-life interventions targeting epigenetic pathways, such as maternal dietary supplementation with folate and omega-3 fatty acids, may mitigate the impact of prenatal stress [18–20]. Importantly, Kallak et al. [21] demonstrated that these epigenetic responses to maternal psychological distress can be sex-specific. Their study identified differential DNA methylation signatures in neonates exposed to prenatal depression and anxiety, suggesting that male and female fetuses may follow distinct biological trajectories in response to similar prenatal stress exposures. This supports the growing recognition of sexually differentiated vulnerability in fetal programming.

## Telomere biology and prenatal stress

Telomeres, DNA–protein structures that protect chromosome integrity, can lead to disease through a number of pathways. It must be underlined that telomeres are sensitive to early-life stress and stress-induced telomere damage can start prenatally. Studies have shown that maternal stress during pregnancy is associated with shorter telomere length in newborns, a potential biomarker of accelerated cellular aging. Moreover, short telomeres can be directly inherited from parental germline [22]. Naude et al. provide information on the relationship between early-life telomere length and prenatal psychological adversities and neurodevelopmental consequences in a population of low-income African mothers and their children [23]. Telomere length has also been studied in populations exposed to major stressors, such as the Great East Japan earthquake (2011), revealing significant reductions in telomere length among children conceived after the disaster [24].

## Psychosocial adversity and neurodevelopment

A paradigm analogous to the Dutch famine can be observed in Romanian orphanages where children subjected to severe neglect exhibited profound neurodevelopmental deficits [25]. Even when physical needs were met, the absence of psychosocial stimulation was deleterious to brain maturation and behavioral adaptation. Long-term follow-ups indicate that the earlier the children are placed in enriched environments, the greater their neurodevelopmental recovery. Specifically, the provision of sensitive and consistent caregiving responsive to a child’s needs is a very important intervention that needs to be readily available for children who are orphaned, abandoned, and maltreated. Such care needs to be individualized and have the best interest of the child in mind.. Early intervention through placement in nurturing, stable environments plays a critical role in mitigating the long-term effects of early emotional deprivation. The results of the review article by Mandl et al. demonstrated structural and functional aberrations in the limbic system, prefrontal cortex, and insula in fetuses and infants prenatally exposed to maternal distress. The prenatal environment shapes the offspring’s phenotype [26]. Deuschle et al. studied 79 pregnant women who underwent amniocentesis in the early second trimester and detected brain-derived neurotrophic factor (BDNF), cortisol, and cortisone in amniotic fluid. It was found that the endocrine data were related to maternal early-life adversities, perceived stress, anxiety, and socioeconomic status. BDNF in amniotic fluid was positively related to maternal early adversity [26, 27]. The study by DiPietro et al. elaborates on a difficult topic, namely, the gestational origins of sex as a moderator of development. Converging evidence confirms that infancy and early childhood developmental outcomes of male fetuses exposed to prenatal and perinatal adversities are more highly impaired than those of female fetuses [25].

## Sex differences in fetal brain development

Wheelock et al. investigated sex differences in fetal brain FC and demonstrated that males exhibited larger prefrontal cortex, amygdala, and hippocampus volumes than females [15]. These volumetric variations may predispose males and females differentially with respect to developmental vulnerabilities and neurodevelopmental disorders.

Steroid hormone receptors are expressed throughout the brain allowing for reciprocal communication between brain and body as well as developmental changes in response to stress, especially during critical periods of plasticity such as during fetal life. Moreover, sex differences exist in many neural and behavioral functions that are developmentally programmed by hormones [8, 9]. A prospective study of mother–child dyads further revealed that elevated maternal cortisol levels in early pregnancy correlated with increased amygdala volume at age 7, but only in females [5]. Additionally, some studies have reported early sex differences in axis activity, which tend to amplify at puberty. Sandman and Davis have shown that the developmental origin of disease or fetal programming predicts that early exposure to adverse conditions have lifelong consequences for health. Fetal exposure to intrauterine conditions, including raised stress hormones, increases the risk for a spectrum of health problems depending on the timing of exposure, the timetable of organogenesis, and the developmental markers used to assess [18].

Of note, a comprehensive review of the literature suggests that sex differences in vulnerability to prenatal stress are not only evident but may be rooted in developmental timing and differential placental responses [28].

Neurodevelopmental programming requires tight regulation of transcriptional processes. During prenatal and postnatal periods, epigenetic processes fine-tune neurodevelopment towards an end-program that determines how an organism interacts with and responds to exposures and experiences throughout life [19]. Notably, the placenta also exhibits sexually dimorphic gene expression patterns, influencing fetal responses to intrauterine stressors [10]. There is good evidence suggesting that the deleterious consequences of exposure to childhood maltreatment do not cover only the individual’s lifespan but may be transmitted across generations [20]. Nevertheless, the mechanisms involved are not apparent.

Recent neuroimaging evidence confirms that maternal psychological distress during pregnancy can lead to structural and functional alterations in the fetal brain, particularly in regions involved in emotion regulation and cognition, such as the hippocampus, prefrontal cortex, and amygdala. These effects appear to be sex-specific, with differential vulnerability across male and female fetuses [29].

## The role of mini-puberty in sexual differentiation and neurodevelopment

An important yet often under-discussed aspect of early hormonal development is the period of “mini-puberty,” a transient postnatal activation of the hypothalamic-pituitary–gonadal (HPG) axis that occurs during the first months of life. This phase is characterized by a temporary surge in sex hormones—testosterone in males and estradiol in females—which plays a critical role in brain sexual differentiation, genital development, and potentially, the early programming of gender identity and neurobehavioral traits [30]. More recent findings further emphasize the physiological significance of mini-puberty as a window of opportunity for early detection and therapeutic intervention in infants with disorders of sex development and congenital hypogonadism [31]. Given that prenatal stress and exposure to endocrine disruptors may impair the function of the HPG axis, disruptions during this critical period may have lasting effects on sex-specific brain development and later psychosocial outcomes. Future research should explore how prenatal adversity interacts with postnatal endocrine events such as mini-puberty to influence long-term developmental trajectories**.**

## Resilience and protective factors

Nolvi et al. examined maternal stress and resilience factors, highlighting the role of postnatal environments in moderating prenatal stress outcomes [32]. High-quality early caregiving and improved socioeconomic conditions were associated with normalized hippocampal and limbic connectivity, underscoring the plasticity of early neurodevelopment. Moreover, maternal social support and prenatal interventions, such as mindfulness-based stress reduction and cognitive-behavioral therapy, have shown promise in mitigating the adverse effects of prenatal stress on offspring neurodevelopment.

## Conclusion

Emerging evidence underscores the profound impact of prenatal stress on fetal brain development, with distinct sex-specific vulnerabilities. The interplay between genetic, epigenetic, hormonal, and environmental factors critically shapes individual trajectories of neurodevelopment and long-term mental health. Advances in neuroimaging, molecular biology, and endocrinology have enabled a more nuanced understanding of how prenatal adversity may affect brain maturation, particularly through mechanisms such as HPA axis dysregulation, telomere shortening, and altered gene expression. Future research should continue to elucidate the precise pathways through which prenatal stress disrupts neurodevelopmental processes, ideally through integrative approaches combining longitudinal designs, neuroimaging, and biological markers. Targeted early interventions—including prenatal counseling, nutritional optimization, and maternal psychosocial support—may help buffer these effects and promote resilience in vulnerable populations.

Importantly, while the reviewed literature provides compelling evidence for associations between prenatal stress and neurodevelopmental alterations, definitive causal relationships have yet to be established. Most available studies are observational and, thus, cannot confirm causality. Clarifying these links will require well-designed prospective and experimental studies that account for confounders and mediating factors. Until then, interpretations should remain cautious, emphasizing correlation rather than causation.

## Data Availability

The data that support the findings of this study are available from the corresponding author upon reasonable request.
